# Quercetin inhibits the metabolism of arachidonic acid by inhibiting the activity of CYP3A4, thereby inhibiting the progression of breast cancer

**DOI:** 10.1186/s10020-023-00720-8

**Published:** 2023-09-14

**Authors:** Huaming Tang, Yuanli Kuang, Wan Wu, Bing Peng, Qianmei Fu

**Affiliations:** 1 Department of Pancreatic Surgery, West China Hospital, Sichuan University, No. 37, Guoxue Lane, Wuhou District, Sichuan Province Chengdu, 610000 People’s Republic of China; 2 Department of General Surgery, Chongqing Kaizhou District People’s Hospital, Chongqing, 400700 People’s Republic of China; 3 Department of Oncology, Chongqing Kaizhou District People’s Hospital, No. 8, Ankang Road, Hanfeng Street, Kaizhou District, Chongqing, 400700 People’s Republic of China

**Keywords:** Saffron, Quercetin, Breast carcinoma, Cytochrome P450 family 3 subfamily A member 4, Arachidonic acid, Epoxyeicosatrienoic acids

## Abstract

**Background:**

Recent years have witnessed impressive growth in applying natural medicine in tumor treatment. Saffron is reported to elicit an inhibitory property against BC. Herein, we sought to explore the specific components and mechanistic basis of saffron’s anti-breast carcinoma (BC) function.

**Methods:**

Bioinformatics analysis was employed to analyze saffron components' anti-BC activity and screen the corresponding target genes involved in BC. Then, the roles of the main saffron ingredient quercetin in the activity of BC cells were examined using CCK-8, MTS, flow cytometry, colony formation, Transwell, and Gelatin zymogram assays. Additionally, the interactions among Quercetin, EET, and Stat3 were assessed by immunofluorescence and Western blot, and LC–MS/MS determined the levels of AA, EETs, and CYP3A. Finally, BC xenograft mouse models were established to verify the anti-BC function of Quercetin in vivo.

**Results:**

Quercetin, the main active component of saffron, inhibited BC progression. Quercetin suppressed BC cell growth, migration, and invasion and inhibited CYP3A4 expression and activity in BC. Mechanistically, Quercetin down-regulated CYP3A4 to block the nuclear translocation of Stat3 by decreasing the metabolization of AA to EETs, thereby alleviating BC. Moreover, exogenously added EETs counteracted the anti-tumor effect of Quercetin on BC. Quercetin also inhibited the tumor growth of tumor-bearing nude mice.

**Conclusion:**

Quercetin could inhibit the activity of CYP3A to down-regulate AA metabolites EETs, consequently hampering p-Stat3 and nuclear translocation, thus impeding BC development.

**Supplementary Information:**

The online version contains supplementary material available at 10.1186/s10020-023-00720-8.

## Background

Breast carcinoma (BC) remains one of the most frequently diagnosed cancers worldwide. It is the primary cause of cancer-associated mortality among women in over 100 countries (Bray et al. 2018). Currently, cancer therapies based on natural nutritional components have gained the attention of many scientists. Saffron, an extract from the *Crocus sativus* flower, is proposed to induce cell apoptosis in multiple types of cancers, such as colorectal, bladder, and pancreatic cancer, thus eliciting its anti-tumor effect in cancers (Bolhassani et al. 2014). However, the properties of saffron components in BC development and the involved pathways demand an extensive investigation.

Essentially, Quercetin has been reported as the main active ingredient of saffron (Sola et al. 2018). Notably, as a member of the flavonoid class enduring the innate apoptosis capacity, Quercetin could radically inhibit the proliferative potential of several human BC-related cell lines (Ezzati et al. 2020). Furthermore, Quercetin competitively inhibits the effects of metabolizing enzyme CYP3A4, thus affecting the metabolism of rivastigmine (Palle and Neerati 2017). Shang et al. reported that CYP3A4 is a crucial CYP450 enzyme in HepG2 cells, accounting for more than 50% of the metabolism of FLX (Prozac) (Shang et al. 2016).

Moreover, epoxyeicosatrienoic acid (EET) is a metabolite of arachidonic acid (AA) (Karkhanis et al. 2017), and EET production is closely associated with the initiation and development of BC (Thuy Phuong et al. 2017). Stat3 is abnormally active in BC and is thus extensively identified as a therapeutic target for various cancers (Segatto et al. 2018). Functionally, CYP3A4 could produce EET by stimulating the metabolism of AA, inducing the nuclear translocation of phosphorylated signal transducer and activator of transcription 3 (Stat3), ultimately aggravating estrogen receptor (ER)-positive BC (Mitra et al. 2011).

As a result, we hypothesized that Quercetin might inhibit CYP3A4 to diminish AA's generation of EETs, preventing the nuclear translocation of p-Stat3 and therefore reducing the development of BC. Using a series of gain- and loss-of-function experiments, we verified the anti-tumor effects of Quercetin on BC in vitro and in vivo.

## Materials and methods

### Ethics statement

The animal experiments were conducted in accordance with the recommendations of the Animal Ethics Committee of Sichuan University (20200714040). Adequate measures were taken to minimize the number of mice and their suffering.

### Bioinformatics analysis

BC-related target genes were identified using the DisGeNET database and the GeneCards database. Next, the traditional Chinese medicine systems pharmacology (TCMSP) database was adopted to retrieve the main components of saffron for extensive conditional screening (OB ≥ 30%, DL ≥ 0.18). Next, the Comparative Toxicogenomics Database (CTD) data (http://ctdbase.org/detail.go? acc = C452899 & type = chem) was used to retrieve the target genes of the main components in saffron. Next, the potential target genes were determined by the intersection of the DisGeNET, GeneCards, and CTD databases concerning the BC-related target genes. Finally, the STRING database was employed to plot the protein–protein interaction network diagram (Szklarczyk et al. 2021).

### Topology and cluster analysis

Cytoscape was applied to plot the network diagram and perform data analysis for screening of the target genes [BC ≥ Avg (BC), CC ≥ Avg (CC), and De ≥ Avg (De)]. Additionally, the Molecular Complex Detection (MCODE) plugin in the Cytoscape software was adopted for cluster analysis of the network diagram (Edge and Compton 2010, Su et al. 2019, Nagtegaal et al. 2020).

### Enrichment analysis of biological targets

The databases for annotation, visualization, and integrated discovery (DAVID) online were employed for the Kyoto Encyclopedia of Genes and Genomes (KEGG) analysis and functional enrichment analysis. In addition, Cytoscape was employed to plot a network diagram of the KEGG analysis results (Huang da et al. 2009).

### Cell culture

In this study, we employed four breast cancer cell lines (ZR-75-1, MCF-7, T47D, and MDA-MB-231) and two normal human breast epithelial cell lines (MCF10A and MDA-kb2), all of which were purchased from Biobw (Beijing, China). Cells were cultured at 37 °C with 5% CO_2_ in L-15 medium, Dulbecco’s modified Eagle’s medium (DMEM), serum-free cell freezing medium (RPMI)-1640, and L-15 culture medium, respectively. The medium contained 10% (v/v) FBS (Gibco, Carlsbad, CA), 100 µg/mL penicillin, and 100 µg/mL streptomycin (Ma et al. 2003; Goh et al. 2022).

### Cell viability detection

MTS assay was used to evaluate cell proliferation. Briefly, the BC cells (ZR-75-1 and MCF-7) were seeded in 96-well plates at 5 × 10^3^ cells/well density. After incubation at 37 °C for 24 h, the cells were supplemented in a medium containing different concentrations of Quercetin (0, 1, 2, 5, and 10 μM). Following incubation for another 48 h, 20 μL Aqueous One Solution was added for 2 h culture, after which the absorbance value at the wavelength of 490 nm was measured using a microplate reader. According to the value of absorbance, the survival of cells was converted into survival rate (cell viability = (mean OD of experimental group-mean OD of blank)/(mean OD of control group-mean OD of the blank group) × 100%) (Dong et al. 2010, Edge and Compton 2010, Nagtegaal et al. 2020). 

### Cell proliferation assay

Cell counting kit (CCK)-8 assay detected cell proliferation. ZR-75-1 and MCF-7 cells were seeded in 96-well plates at a 5 × 10^3^ cells/well density. After incubation at 37 °C for 24 h, the cells were added to the culture medium in the presence of different concentrations of Quercetin (0, 1, 2, 5, 10 μM). After continuing the culture for 48 h, each well was added with 100 μL of CCK8 solution, incubated for about 1 h in the culture, and then the absorbance was measured at 450 nm with a microplate reader. The absorbance value OD450 was used to evaluate the ability of cell proliferation (Adan et al. 2016, Chen et al. 2020).

### Cell cycle analysis

Quercetin was purchased from Sigma (St. Louis, MO) in the experiment. Quercetin was insoluble in water (practically insoluble). DMSO was added as the solvent to dissolve Quercetin according to the instructions. ZR-75-1 and MCF-7 cells were treated with designated concentrations of Quercetin (0, 2, or 5 μM) for 24 h and trypsinized. Following centrifugation at 800 rpm for 5 min, the collected cells were resuspended using 0.3 mL phosphate buffer saline (PBS) to prepare a single-cell suspension, which was then added into 0.7 mL of pre-cooled anhydrous ethanol and fixed at 4 °C overnight. After centrifugation and two PBS rinses, the fixed cells were resuspended in 500 μL PBS supplemented with RNase A (100 μg/mL) and propidium iodide (PI, 50 μg/mL) solution and incubated at 37 °C for 30 min in conditions devoid of light. The distribution of the cell cycle was analyzed using a flow cytometer (Vartholomatos et al. 2015).

### Cell apoptosis analysis

For apoptosis detection, ZR-75-1 and MCF-7 cells were treated with designated concentrations of Quercetin (0, 2, or 5 μM) for 24 h and trypsinized. Following centrifugation at 800 rpm for 5 min, the collected cells were resuspended using 0.3 mL PBS to make a single-cell suspension. Next, the cells were detached and resuspended using 0.1 mL 1 × binding buffer (BD Biosciences, Pasadena, CA) and incubated with PI and Annexin V for 30 min in conditions devoid of light. Cell apoptosis was also assessed using a flow cytometer (Li et al. 2015).

### Colony formation assay

Tumor cells (ZR-75-1 and MCF-7 cells; approximately 1 × 10^3^ cells/well) were inoculated in 6-well plates. After 24 h of culture, a fresh medium with different concentrations of Quercetin (0, 2, or 5 μM) was added to the wells. After incubation for another 8 days, 4% paraformaldehyde was added to fix cells and stained with 0.1% crystal violet (Dong et al. 2019).

### Cell adhesion assay

The ZR-75-1 and MCF-7 cells were treated with different concentrations of Quercetin (0, 2, or 5 μM) for 12 h, followed by trypsinization and counting. Then, the cells (10^5^ cells/well) were inoculated in 96-well plates pre-plated with fibronectin and supplemented with a quercetin of different concentrations (0, 2, or 5 μM). After incubation at 37 °C for 25 min, the adhered cells were stained with 0.1% crystal violet. Following PBS rinse, the crystal violet in stained cells was dissolved using 30% ice acetic acid, and the absorbance value at 590 nm was read using a microplate reader (Pehlivanova et al. 2012).

### Western blot analysis

Cells were lysed with the radioimmunoprecipitation assay buffer containing 150 mM sodium chloride, 1% Triton X-100, 0.5% sodium deoxycholate, 0.1% sodium dodecyl sulfate, 50 mM Tris, and a mixture of protease and phosphatase inhibitors. First, bicinchoninic acid (BCA) kit (Thermo) was used to determine the concentration of the extracted protein. Next, the protein was separated by 8–15% sodium dodecyl sulfate–polyacrylamide gel electrophoresis and then electrotransferred onto a polyvinylidene fluoride membrane. Then, a membrane blockade was conducted using 5% bovine serum albumin (BSA) and incubated with corresponding primary antibodies to CYP3A4 (ab230022, Abcam), PCNA (ab92552, Abcam), p21 (ab109199, Abcam), caspase3 (ab32351, Abcam), cleaved caspase3 (ab2302, Abcam), PARP (9542, Cell Signaling Technology, Shanghai, China), cleaved-PARP (ab32064, Abcam), β-actin (ab6276, Abcam), Stat3 (ab119352, Abcam), p-Stat3 (ab76315, Abcam), matrix metalloproteinase 2 (MMP2) (ab181286, Abcam), MMP9 (ab76003, Abcam), TIMP1 (ab211926, Abcam), and TIMP2 (ab180630, Abcam). Following three rinses with Tris Buffered Saline Tween (TBST), the membrane was incubated with the horseradish peroxidase (HRP)-labeled anti-rabbit or anti-mouse secondary antibody (Sigma). After another three TBST rinses, the LI-COROdyssey System was employed for membrane scanning and image analysis (Rana et al. 2019).

### Transwell assay

Boyden chambers consisting of an apical chamber and a basolateral chamber (with a well size of 8 μm) were employed, which can be placed in a 24-well plate.

For detecting cell migration, the cells were detached, counted, and prepared into cell suspensions containing different concentrations of Quercetin (0, 2, or 5 μM) (ZR-75-1: 50,000/100 μL, MCF-7: 100,000/100 μL), 100 μL of which were added to the apical chamber, whereas 600 μL of Quercetin with the corresponding concentration was added to the basolateral chamber. After about 10 h of incubation at 37 °C, the chamber was fixed using paraformaldehyde for 15 min and stained with 0.1% crystal violet. Then the cells were photographed and counted. Finally, the chamber was pre-coated with Matrigel in the cell invasion assay, and the remaining steps were the same as mentioned above (Pan et al. 2011).

### Immunofluorescence

The coverslip of tumor cells was placed in a 24-well plate and incubated with 0.2% gelatin for 30 min. ZR-75-1 cells were inoculated on the gelatin-coated coverslip. Upon attaining 70% cell confluence, the cells were treated with different concentrations of Quercetin and then removed from the medium, followed by 15 min fixation with 4% paraformaldehyde. Following three rinses, the cells were treated with 0.1% TritonX-100 for 30 min. Then, the cells were treated with 1% BSA for 30 min and subject to overnight incubation with the corresponding primary antibodies [paxillin (ab32084), ac-tubulin (ab179484), and p-Stat3 (ab76315)] at 4 °C followed by three rinses with phosphate-buffered saline with tween detergent (PBST). Next, the cells were incubated with the corresponding fluorescent secondary antibody for at least 1 h (depending on the microscopy results) and rinsed 3 times with PBST. Following a 3-min staining regimen with 4′,6-diamidine-2-phenylindole (DAPI) for nuclear labeling and three rinses with PBST, the coverslip was placed upside down on a glass slide supplemented with the mounting solution and dried with filter paper. Finally, the coverslip was sealed with nail polish and photographed under a confocal laser microscope (Confocal) (Huang et al. 2021).

### Effects of Quercetin on the activity of MMPs by using gelatinase enzyme spectrum analysis

ZR-75-1 cells were seeded in a culture dish with a diameter of 6 cm. When the cell density reached approximately 80%, the culture medium was aspirated, and the cells were washed twice with PBS. Next, the cells were cultured in serum-free MEM medium with 2 μM and 5 μM Quercetin for 24 h. After Quercetin treatment for 24 h, the cells were collected, and their protein extracts were analyzed to detect the expression of MMP2 and MMP9 activity-related proteins. Electrophoresis was conducted in non-reducing conditions using a 10% sodium dodecyl sulfate–polyacrylamide gel (SDS-PAGE) containing 1% type I bovine skin gelatin. Pre-stained SDS-PAGE standards (BIO-RAD Laboratories Ltd., Hercules, CA, USA) were used as low molecular weight markers. The gel was run at 120V/25A, then incubated in buffer I (50 mmol/L Tris HCl, 2.5% Tween 80, 0.02% NaN3(w/v), pH 7.5) at 22 °C for 30 min. Next, the gel was further incubated in buffer II (50 mmol/L Tris HCl, 2.5% Tween 80, 0.02% NaN3 (w/v), 1μM ZnCl2 and 5 mmol/L CaCl2 at 22 °C) for 30 min. After that, the gel was incubated in buffer III (50 mmol/L Tris HCl, 5 mmol/L CaCl2, 1μM ZnCl2, and 0.02% NaN3(w/v) at 37 °C) for 12 h. Next, the gel was stained with 0.1% Coomassie Brilliant Blue R-250 at 22 °C for 60 min and then for 30 min in a solution containing 10% acetic acid and 10% methanol (Zarella et al. 2016, Tajhya et al. 2017). Finally, the gel was scanned (Image scanner, Amersham Biosciences, Uppsala, Sweden), and the band intensity was quantified using Kodak molecular imaging software (version 4.5, Kodak, Rochester, NJ, USA).

### Measurement of CYP3A activity, AA and EETs

ZR-75-1 and MCF-7 cells were inoculated into a 12-well plate (ZR-75-1: 100,000/200 μL, MCF-7: 200,000/200 μL). After attaining 80–90% confluence, the cells were treated with a complete medium containing different concentrations of Quercetin (0, 2, or 5 μM) for 2 h. Afterward, the cells were cultured with a complete medium containing the CYP3A probe substrate midazolam (10 μM) for another 2 h, detached using trypsin, and resuspended in deionized water. The cells were disrupted after treatment with repetitive freezing and thawing (three times) and ultrasound (3 s sonication and 10 s pause on ice, 30 cycles). The AA and its metabolites EETs in the cell sap were extracted with ethyl acetate, the organic extracts were saponified, the obtained fatty acids were extracted into the acidified ether and evaporated under N_2_, and the samples were dissolved in MeOH for LC–MS/M mass spectrometry analysis (Jabor et al. 2005).

For the detection of AA and its metabolite EETs in the tissue, 100 mg of the sample was taken out, added with 10 times the volume of pre-cooled 90% methanol (addition of 1 mL of 90% methanol to 100 mg sample), frozen with liquid nitrogen, and homogenate crushing was performed using MP homogenizer (24 × 2, 6.0 M/S, 20 s, 3 times), followed by low-temperature ultrasound 30 min/time for 2 times. Then, the samples were placed at – 20 °C for 60 min, centrifuged at 13,000 g at 4 °C for 15 min, and the supernatant (divide) was taken and aliquoted as 900 μL/tube), vacuum dried, freeze-dried powder, and stored at – 80 °C for later use. Next, 100 μL acetonitrile aqueous solution (acetonitrile: water = 1:1, v/v) was added to reconstitute during mass spectrometry, vortexed, centrifuged at 14,000 g at 4 °C for 15 min. Finally, the supernatant was taken for sample analysis, and for measurement of AA and EETs, the AA and its metabolites EETs in the cell fluid extracted with ethyl acetate were analyzed by LC–MS/MS, which was described previously (Mitra et al. 2011).

### Xenograft models in nude mice

Four-week-old male BALB/c nude mice (Beijing HFK Bioscience Co., Ltd, Beijing, China) were raised in a specific pathogen-free animal house one week before acclimatization to the breeding environment. Meanwhile, ZR-75-1 cells were resuspended (5 × 10^7^ cells/mL), and 100 μL of the suspension was randomly injected into the right dorsal of each nude mouse. When the average tumor volume reached 50 mm^3^, the nude mice with no significant difference in tumor volume were randomly divided into a control group and an administration group (low-dose, high-dose). The control group was subjected to intraperitoneal injection with 0.5% CMC-Na (drug solvent, 1 mL/kg/day). In contrast, the administration group was subjected to intraperitoneal injection with Quercetin (20 mg/kg/day, 40 mg/kg/day). The tumor volume (TV) was measured daily and calculated using the formula: TV = A × B^2^ × 0.52, where A is the longest diameter of solid tumors (mm). B is the shortest diameter of solid tumors (mm). Finally, tumors excised from the sacrificed mice were photographed, weighed, and fixed. All experiments were conducted blindingly (Ling et al. 2008; Wang et al. 2014).

### Hematoxylin–eosin (HE) staining

The tumors and visceral tissues of the mice after quercetin treatment were fixed using 4% formaldehyde overnight. Then the paraffin-embedded tissues were sectioned. Subsequently, the sections were stained with hematoxylin, and hydrochloric acid-alcohol was added to separate the color for 20–30 s. After providing a blue gamut, the sections were re-stained with 0.5% eosin for 5 s, cleared by xylene, and sealed (Tang et al. 2014). Finally, the sections were observed, pictured, and analyzed using a microscope and the Pro Plus software.

### Statistical analysis

The statistical analysis was performed using the SPSS 21.0 statistical software. Measurement data were summarized as the mean ± standard deviation. The unpaired *t*-test was adopted for comparing data between groups, and the one-way analysis of variance (ANOVA) or repeated measures ANOVA was adopted for comparison between multiple groups, followed by Tukey’s post hoc test (Tang et al. 2014). A value of* p* < 0.05, *p* < 0.01, *p* < 0.001, or *p* < 0.0001 indicated that the difference was statistically significant.

## Results

### Analysis of the main components of saffron and preliminary screening of its anti-breast cancer activity monomers

**Fig. 1:**
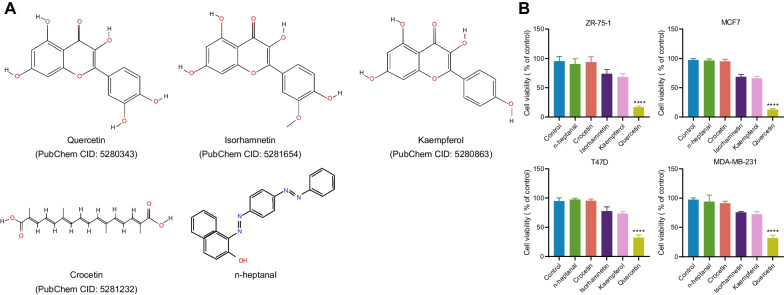
Main components of saffron. **A** The structural formula of the main components of saffron. **B** Screening for the anti-breast cancer activity of the main components of saffron against four types of breast cancer. (The data results are expressed in mean ± standard deviation for quantitative data. One-way analysis of variance was used for multiple group comparisons, followed by Tukey’s post hoc test. The cell experiments were repeated 3 times. **** indicates that the difference is statistically significant compared to the Control group with a P < 0.0001)

In this study, bioinformatics analysis was adopted to analyze and screen the main components of saffron. Seventy kinds of saffron components were identified through the TCMSP database. By consulting references, we screened out several candidate factors that inhibit cell proliferation, which was verified by in vitro experiments, and five of them were finally chosen (Fig. 1A), including n-heptanal (not included in PubChem database), Crocetin (PubChem CID: 5281232), isorhamnetin (PubChem CID: 5281654), kaempferol (PubChem CID: 5280863), and Quercetin (PubChem CID: 5280343).

First, the saffron extract was used to treat BC cells (Additional file 1: Figure S1), whose results uncovered that saffron had a certain inhibitory function on the growth of BC cells. To explore the anti-BC activity of saffron and the main components of it which possess its anti-cancer activity, this study examined the effects of those mentioned above five main components on the proliferation of BC cells. The preliminary screening results from MTS revealed that ZR-75-1 and MCF7 cells showed better sensitivity to Quercetin (Fig. 1B).

To further determine the effects of the main active ingredients, namely Crocetin, isorhamnetin, kaempferol, n-heptanal, and Quercetin, on the cytotoxicity of breast cancer MCF-7 and MDA-MB-231 cells, different concentrations of Crocetin, isorhamnetin, kaempferol, and Quercetin were used to treat breast cancer MCF-7 and ZR-751 cell lines, and the effects of different drug concentrations on cell toxicity and activity were tested. The results showed that Quercetin had a significant cytotoxic effect on breast cancer cells at a low dose (1 μM) (Additional file 2: Figure S2A-E). Meanwhile, we also tested the cytotoxicity of Quercetin on normal human breast cells MCF10A and MDA-kb2, and the results showed that 20 μM Quercetin had a significant cytotoxic effect on normal breast cells (Additional file 2: Figure S2F). Therefore, we speculate that Quercetin may be the main monomer that plays a role in the saffron extract. Therefore, in the subsequent experiments, we chose to study Quercetin to explore further its mechanism in combating breast cancer.

### Quercetin inhibits BC cell growth

**Fig. 2:**
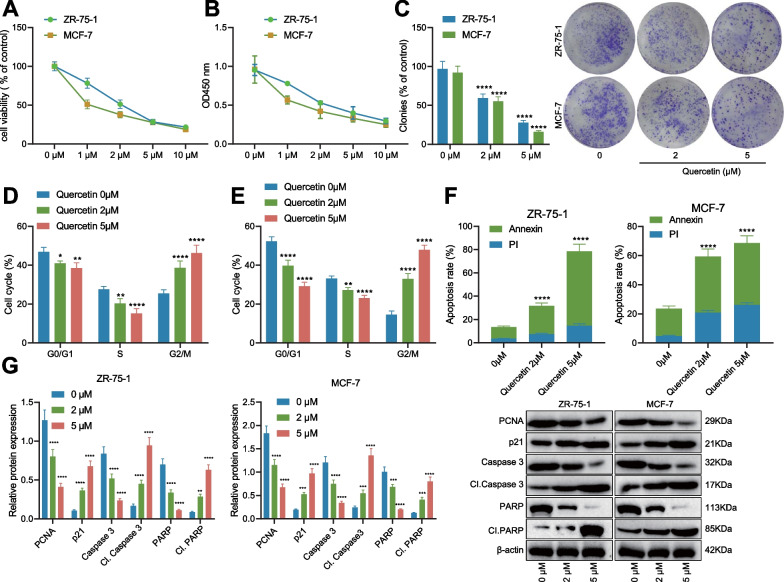
Effects of quercetin on proliferation and apoptosis of breast cancer cells. **A** MTS assay was used to determine the viability of breast cancer cells in each group; **B** CCK-8 assay was used to determine the proliferation of breast cancer cells in each group; **C** colony formation assay was used to determine the colony formation ability of breast cancer cells in each group; **D**, **E** flow cytometry was used to determine the cell cycle of breast cancer cells in each group; **F** flow cytometry was used to determine the apoptosis of breast cancer cells in each group; **G** Western blot was used to determine the protein expression of proliferation- and apoptosis-related factors in breast cancer cells of each group. The data results are represented as mean ± standard deviation for quantitative data. Independent-sample t-test was used to analyze two groups, one-way ANOVA was used to analyze multiple groups, and Tukey’s post hoc test was used for comparison. The cell experiments were repeated 3 times. * indicates P < 0.05 compared with the Quercetin 0 μM group, ** indicates P < 0.01, *** indicates P < 0.001, and **** indicates P < 0.0001

To further verify the effect of Quercetin on tumor cell proliferation and apoptosis, we adopted MTS to detect the viability of tumor cells. After treatment with Quercetin, the viability of ZR-75-1 and MCF-7 cells was inhibited in a concentration-dependent manner, and the half-maximal inhibitory concentration IC_50_ was 2.46 μM and 1.01 μM, respectively (Fig. 2A). CCK8 assay was performed to detect the proliferation of tumor cells, which revealed that Quercetin significantly inhibited the proliferation of ZR-75-1 and MCF-7 cells (Fig. 2B). The colony formation assay results unraveled that Quercetin had a significant inhibitory effect on the clone formation of ZR-75-1 and MCF-7 cells (Fig. 2C). Then, the flow cytometry results showed that Quercetin blocked the cell cycle of ZR-75-1 and MCF-7 cells at the G2/M phase in a dose-dependent manner (Fig. 2D, E). Besides, Quercetin significantly induced the apoptosis of ZR-75-1 and MCF-7 cells (Fig. 2F). Western blot analysis confirmed that Quercetin significantly reduced PCNA (a marker protein for proliferation) and cyclin p21 expression patterns but increased PARP (a marker protein for apoptosis) and caspase 3 expression patterns as well as the expression patterns cleaved-PARP and cleaved-caspase 3 (Fig. 2G). Coherently, Quercetin inhibited the growth of BC cells while promoting cell apoptosis.

### Quercetin restrains the adhesion, migration, and invasion of BC cells

**Fig. 3:**
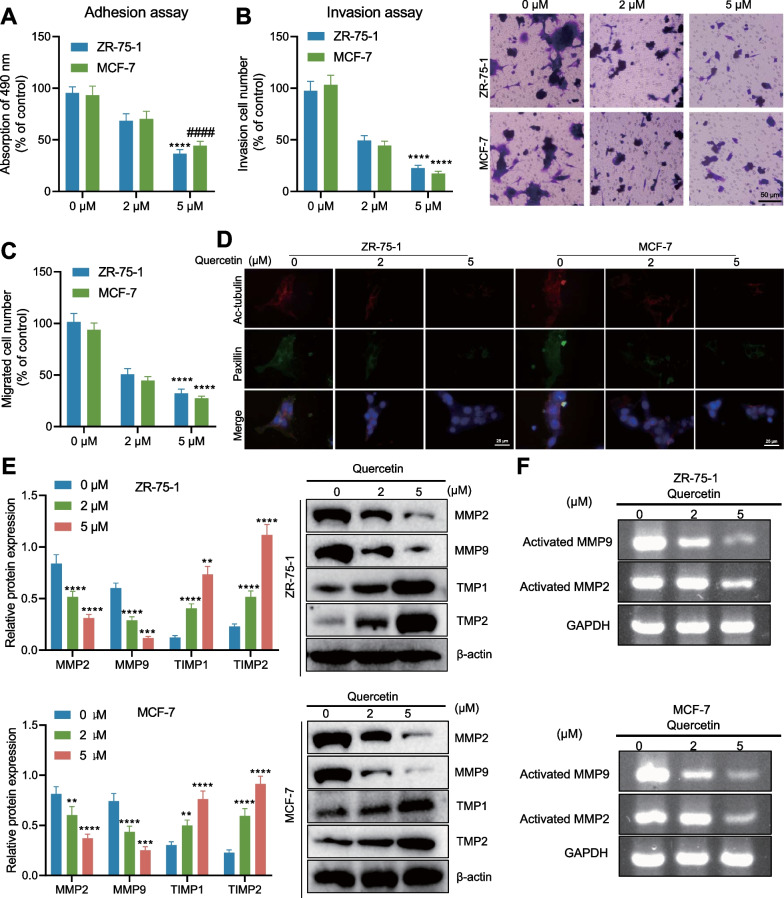
The effect of Quercetin on the adhesion, migration, and invasion of breast cancer cells. **A** Cell adhesion experiment detecting the adhesion of different groups of breast cancer cells; **B** Transwell experiment detecting the invasion ability of different groups of breast cancer cells; **C** Transwell experiment detecting the migration ability of different groups of breast cancer cells; **D** Immunofluorescence detects the expression of paxillin and Ac-tubulin in different groups of breast cancer cells; **E** Western blot examine the protein expression of MMP2, MMP9, TIMP1, and TIMP2 in different groups of breast cancer cells; **F** Gelatin zymography analyses the protein expression of activated MMP2 and MMP9 in different groups of breast cancer cells. (The data results are quantitative and represented by mean ± standard deviation. An independent sample t-test was used for analysis between two groups, and a one-way analysis of variance was used for analysis among multiple groups. Tukey’s test was used for post-hoc analysis. The cell experiment was repeated 3 times, *** represents P < 0.001 compared to Quercetin 0 μM, and **** represents P < 0.0001.)

The effects of Quercetin on the adhesion, migration, and invasion of BC cells were also investigated. Cell adhesion and Transwell assay results revealed that Quercetin significantly inhibited the adhesion, migration, and invasion abilities of ZR-75-1 and MCF-7 cells (Fig. 3A–C). Furthermore, we ascertained that Quercetin significantly decreased the expression patterns of focal-adhesion markers paxillin and Ac-tubulin, suggesting that Quercetin could significantly inhibit the adhesion ability of ZR-75-1 and MCF-7 cells (Fig. 3D). Additionally, Quercetin restrained the MMP2 and MMP9 expression patterns but increased the expression patterns of their natural inhibitors, TIMP1 and TIMP2 (Fig. 3E). Further, gelatin zymogram assay revealed that Quercetin noticeably reduced the enzymatic properties of MMP2 and MMP9 compared with 0 μM quercetin treatment (Fig. 3F). Quercetin significantly inhibited the adhesion, migration, and invasion of BC cells.

### Information on biological targets of Quercetin for BC

To further identify the molecular mechanism of Quercetin for inhibiting the growth of BC cells, we initially analyzed the potential target genes of Quercetin through bioinformatics analysis. The DisGeNET and GeneCards databases identified 4962 and 11,187 target genes related to BC, respectively. Besides, 4249 BC-related target genes were obtained through the intersection. Additionally, 4129 target genes associated with Quercetin were retrieved from the CTD database. Through screening the overlapping target genes in DisGeNET, GeneCards, and CTD databases, we identified 1567 quercetin-related target genes in BC (Additional file 3: Figure S3A). By analyzing the network diagram of Quercetin’s target genes relevant to BC, 282 potential target genes were screened (Additional file 3: Figure S3B).

Perform clustering analysis on the network relationship map of Quercetin in 282 potential target genes in breast cancer. The clustering analysis results showed a total of six clusters that were closely related: Cluster 1: score = 69.934, Nodes = 92, Edges = 3182 (Additional file 4: Figure S4A); Cluster 2: score = 27.481, Nodes = 78, Edges = 1058 (Additional file 4: Figure S4B); Cluster 3: score = 6.065, Nodes = 32, Edges = 94 (Additional file 4: Figure S4C); Cluster 4: score = 5.933, Nodes = 31, Edges = 89 (Additional file 4: Figure S4D); Cluster 5: score = 4.500, Nodes = 5, Edges = 9 (Additional file 4: Figure S4E); Cluster 6: score = 3.222, Nodes = 19, Edges = 29 (Additional file 4: Figure S4F). In addition, the KEGG analysis results of these six clusters show that four of them have Pathways in cancer (KEGG: map05200), HTLV-I infection (KEGG: map05166), Viral carcinogenesis (KEGG: map05203), Estrogen signaling pathway (KEGG: map04915), Progesterone-mediated oocyte maturation (KEGG: map04914) and Cell cycle (KEGG: map04110). These six pathways are the main signaling pathways for treating breast cancer with saffron.

The network relationship between these six signal pathways and target genes was plotted (Additional file 3: Figure S3C), and the figure displayed that KRAS, PIK3CA, PIK3R1, and PRKACA were evident in the pathways in cancer, HTLV-I infection, Viral carcinogenesis, Estrogen signaling pathway, and Progesterone-mediated oocyte maturation. Therefore, these four were regarded as the vital target genes for Quercetin in treating BC (Additional file 3: Figure S3D).

### Quercetin retards the metabolism of CYP3A4 in BC cells

**Fig. 4:**
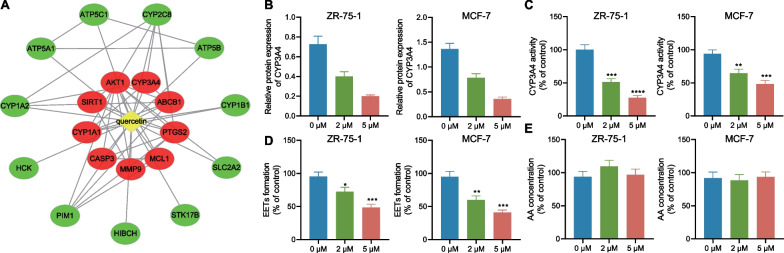
Quercetin affects AA metabolism by regulating the expression and activity of CYP3A4 in breast cancer cells. **A** Potential targets of Quercetin screened from CTD and STITCH databases, with inner circle red genes representing common targets between CTD and STITCH databases, and outer circle green representing targets selected only from CTD database; **B** Western blot detection of CYP3A4 protein expression in various breast cancer cell groups treated with Quercetin; **C** Detection of CYP3A4 activity in various breast cancer cell groups using Midazolam as the probe substrate; **D** LC–MS/MS detection of EETs content in various breast cancer cell groups; **E** LC–MS/MS detection of AA content in various breast cancer cell groups. The results are expressed as means ± standard deviation and analyzed by independent sample t-test for two groups and one-way ANOVA with Tukey’s post-hoc test for multiple groups. The experiments were repeated three times, and * indicates P < 0.05 compared with the Quercetin 0 μM group, ** indicates P < 0.01, *** indicates P < 0.001, and **** indicates P < 0.0001

To further screen targets, we used the STITCH database (http://stitch.embl.de/). The first 20 interaction targets and network diagrams of Quercetin were obtained (Fig. 4A), among which 8 targets were common targets (AKT1, PTGS2, ABCB1, MMP9, CYP3A4, MCL1, CYP1A1, CASP3, and SIRT1) of CTD database and STITCH database. Further KEGG analysis revealed that the estrogen signaling pathway is important in the quercetin regulation of BC, especially ER-positive BC. It has been reported that CYP3A4 can inhibit the growth of ER-positive BC by promoting AA metabolism to produce EETs and then promoting the nuclear translocation of phosphorylated STATE3. Quercetin strongly inhibited CYP3A4 (Mitra et al. 2011; Ostlund et al. 2017; Palle and Neerati 2018; Rasmussen et al. 2017). Based on the results of literature reports and network pharmacology analysis, this study aims to explore the anti-cancer effect and mechanism of quercetin-targeted inhibition of CYP3A4 on BC by inhibiting the metabolism of AA.

To validate whether Quercetin could inhibit the growth of BC through CYP3A4, we adopted Western blot analysis, which revealed that Quercetin could inhibit the CYP3A4 protein expression pattern in the ZR-75-1 and MCF-7 cells in a concentration-dependent manner (Fig. 4B). We further investigated the validity of Quercetin’s suppressive role in CYP3A4 expression in BC cells regarding the activity of CYP3A4. Our test results unraveled that quercetin treatment could significantly reduce the production of 1-hydroxy Midazolam metabolized by CYP3A4 (Fig. 4C). The overhead results supported that quercetin treatment significantly reduced the CYP3A4 expression pattern in the BC cells and inhibited its metabolic activity.

Furthermore, this study explored whether Quercetin’s inhibitory role in CYP3A4 will affect its role in the metabolism of AA in the ZR-75-1 and MCF-7 cells. The LC–MS/MS results showed that quercetin treatment of ZR-75-1 and MCF-7 cells markedly reduced the production of AA metabolites EETs but had no significant effect on the AA concentration in the cells (Fig. 4D, E). Thus, Quercetin essentially suppressed the metabolic activity of CYP3A4, thereby reducing the production of EETs.

### Quercetin reduces p-Stat3 and its nuclear translocation

**Fig. 5:**
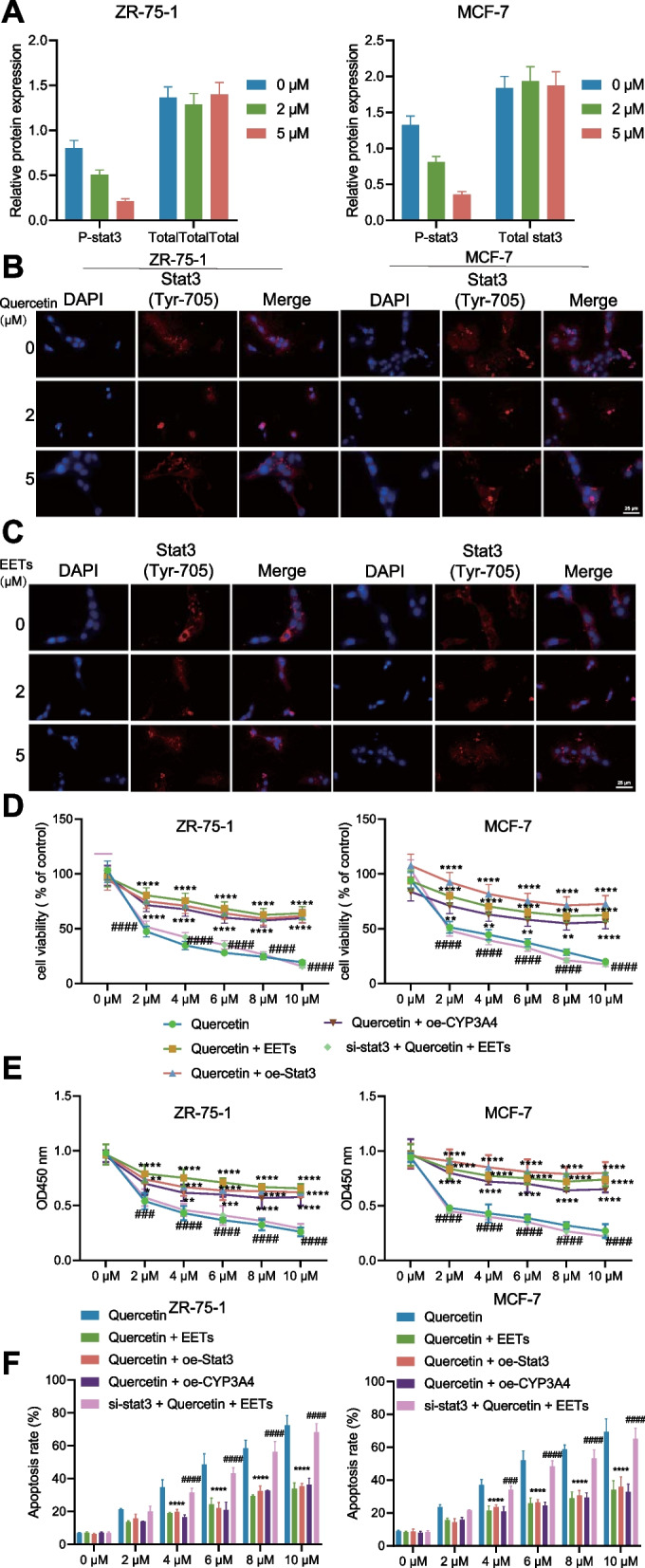
Quercetin regulates the phosphorylation and nuclear translocation of Stat3 through EETs in breast cancer cells. **A** Western blot detects the expression of Stat3 and p-Stat3 (Tyr-705) in each group of breast cancer cells; **B** Immunofluorescence detects the nuclear translocation of p-Stat3 (Tyr-705) in each group of breast cancer cells treated with Quercetin; **C** Immunofluorescence detects the nuclear translocation of p-Stat3 (Tyr-705) in each group of breast cancer cells treated with EET, indicates P < 0.01 compared to the 0 μM group, indicates P < 0.0001; **D** MTS assay detects the cell viability of each group of breast cancer cells; **E** CCK8 assay detects the proliferation ability of each group of breast cancer cells; **F** Flow cytometry detects the apoptosis rate of each group of breast cancer cells, **indicates P < 0.0001 compared to the Quercetin group, ^####^indicates P < 0.0001 compared to the Quercetin + EET group. The data are expressed as mean ± standard deviation for quantitative data, analyzed by independent sample t-test between two groups and one-way ANOVA followed by Tukey’s post-hoc test between multiple groups. Cell experiments are repeated three times

Accumulating evidence suggests that inhibition of CYP3A4 could inhibit p-Stat3 and Stat3 (tyrosine (Tyr)-705) phosphorylation could induce dimerization and nuclear translocation, thus ultimately regulating the expression patterns of several growth-related genes (Mitra et al. 2011). Thus, we tried to explore whether CYP3A4 exerted the same effect on Stat3 in the ZR-75-1 and MCF-7 cells, and Western blot analysis revealed that quercetin treatment reduced the p-Stat3 and had no effect on the total expression of Stat3 (Fig. 5A). Besides, immunofluorescence revealed that quercetin treatment could reduce the nuclear translocation of p-Stat3 (Tyr-705) in ZR-75-1 and MCF-7 cells (Fig. 5B). This result indicates that Quercetin can inhibit the phosphorylation and nuclear translocation of Stat3 in breast cancer cells. Based on the initial results, Quercetin reduced the activity of CYP3A4, thereby inhibiting the production of EETs by AA via CYP3A4. Furthermore, the reduction in intracellular EETs impeded the nuclear translocation of p-Stat3 (Tyr-705) through decreasing p-Stat3 Tyr-705.

To further confirm the preceding conclusions, this study explored the reversal effect of exogenously added 14,15-EET on Quercetin's suppressive role in BC and the effect of Stat3. Immunofluorescence revealed that adding 14,15-EET could significantly promote the nuclear translocation of p-Stat3 (Tyr-705) in ZR-75-1 and MCF-7 cells (Fig. 5C). As reflected by MTS and CCK-8 assays, the promoting effect of Quercetin on the cell viability and proliferation of ZR-75-1 and MCF-7 cells was reversed by EET treatment, overexpression of Stat3 or CYP3A4. At the same time, the knockdown of Stat3 significantly decreased the cell viability and proliferation in the presence of Quercetin and EET. Meanwhile, we knocked down Stat3 on ZR-75-1 and MCF-7 cells, respectively (Fig. 5D, E). Flow cytometry revealed that EET treatment and overexpression of Stat3 or CYP3A4 reduced cell apoptosis of ZR-75-1 and MCF-7 cells. In the presence of Quercetin and EET, an opposite trend was observed after the knockdown of Stat3 (Fig. 5F). Altogether, Quercetin could inhibit the viability and proliferation of BC cells through Stat3, thus exhibiting the vitality of Stat3 in the signaling pathway of 14,15-EET to promote tumor cell growth.

### Quercetin inhibits the tumor growth of BC-bearing nude mice

**Fig. 6:**
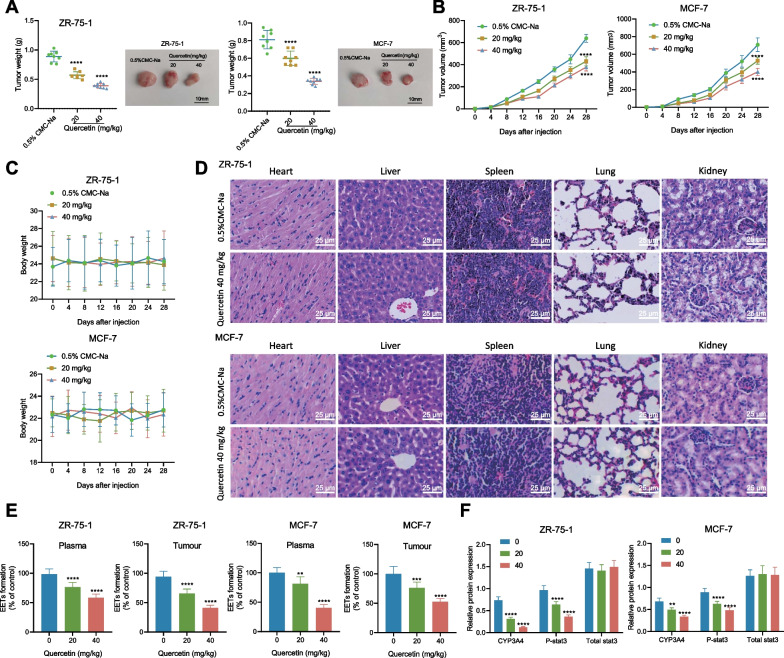
Quercetin affects tumor growth in breast cancer-bearing nude mice. **A**, **B** Tumor size (**A**) and volume (**B**) in each group of tumor-bearing mice; (**C**) the weight of each group of tumor-bearing mice; (**D**) H&E staining detects the pathological conditions of major organs in each group of tumor-bearing mice; (**E**) LC–MS/MS measures the content of EETs in the blood and tumor tissue of each group of tumor-bearing mice; **F** Western blot detects the protein expression levels of CYP3A4, Stat3, and p-Stat3 in tumor tissue of each group of tumor-bearing mice. The data results are metric data represented using mean ± standard deviation. The independent sample t-test was used to compare two groups, and one-way ANOVA was used to analyze the comparison among multiple groups with Tukey’s post-hoc test. Each group had 8 experimental animals, and **** indicates a P value < 0.0001 when compared with the Quercetin 0mg/kg group or 0.5% CMC-Na group

To validate the anti-breast carcinoma effect of Quercetin in vivo, we further verified it in BC xenograft mouse models by subcutaneously injecting ZR-75-1 and MCF-7 cells into nude mice, followed by quercetin injection. Tumor size and volume of tumor-bearing mice were recorded, which revealed that the injection of Quercetin at 20 mg/kg and 40 mg/kg could significantly reduce the tumor size and volume in a dose-dependent manner (Fig. 6A, B). To further investigate Quercetin’s effects on mice’s health status, this study documented and compared changes in body weight after quercetin administration. No significant change was observed in the weight of the quercetin-treated mice treated with different doses of Quercetin (Fig. 6C). Besides, the HE results revealed that the quercetin-treated mice’s heart, liver, spleen, lung, and kidney elicited no considerable pathological changes (Fig. 6D). The results further demonstrated the efficacy and safety of Quercetin against BC in vivo.

To determine the mechanism for the anti-tumor property of Quercetin in vivo, we analyzed and compared the EETs levels in the blood and tumor tissues of the mice after drug administration using LC–MS/MS. The results showed that Quercetin not only reduced the content of EETs in the blood and tissues of the BC-bearing mice (Fig. 6E). Moreover, Quercetin could remarkably reduce the CYP3A and p-Stat3 expression pattern in the tumor tissues (Fig. 6F). In addition, Quercetin elicited the anti-tumor property consistently in the tumor-bearing nude mice and cells.

## Discussion

**Fig. 7:**
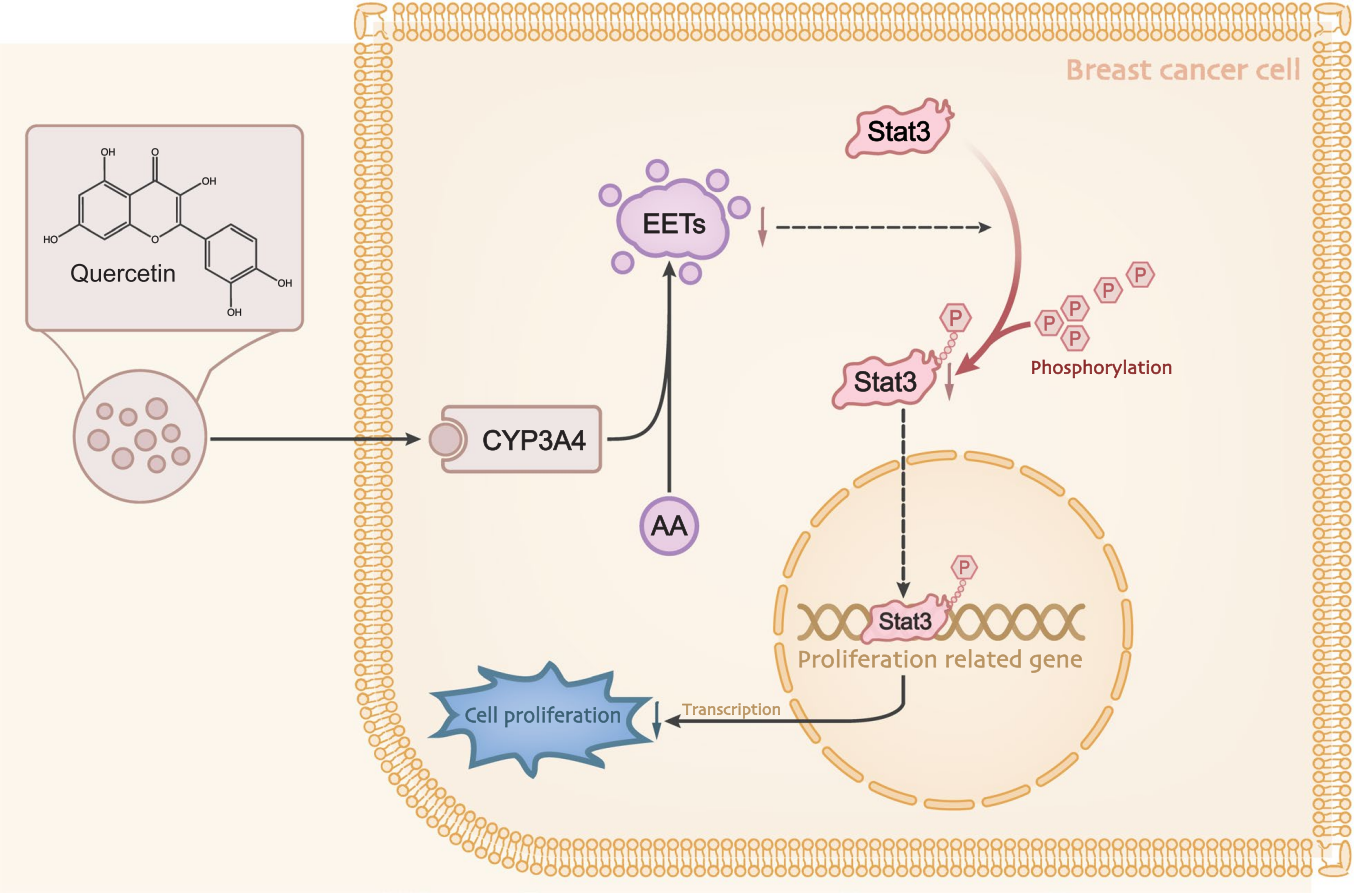
Molecular mechanism schematic diagram of Quercetin inhibiting the metabolism of arachidonic acid and thus inhibiting the progression of breast cancer by inhibiting CYP3A4 activity. Quercetin can reduce the expression of cell proliferation marker protein PCNA, significantly inhibit the expression of cell cycle protein p21, and significantly up-regulate the expression of PARP (cell apoptosis marker protein) and caspase3. At the same time, it also has an up-regulating effect on the expression of cleaved-PARP and cleaved-caspase3 markers of cell apoptosis. Quercetin inhibits the migration, invasion, and adhesion of breast cancer cells. Mechanistic studies indicate that Quercetin can inhibit the activity of CYP3A4 in breast cancer cells, reduce the production of EETs, and the decrease in intracellular EETs content can inhibit the phosphorylation and nuclear translocation of Stat3 (Tyr-705), inhibiting tumor cell growth. Quercetin may inhibit tumor growth by regulating the PCNA/p21 and PARP/caspase3 pathways

BC is a multifaceted heterogeneous disease from the perspective of histological types, treatment responses, metastasis, and outcomes of patients (Prat and Perou 2011). In recent years, increasing natural products have been adopted as suppressors for tumorigenesis (Greenlee 2012). Saffron extracts are proposed to play an inhibitory role in developing papilloma, soft connective tissue sarcoma, and squamous cell carcinoma in mice (Bolhassani et al. 2014), suggesting its anti-tumor properties in cancer. Indeed, existing literature has highlighted the potential of Quercetin to serve as complementary or alternative medicine in BC (Ezzati et al. 2020). This current study elaborates on the mechanistic actions of the anti-tumor effect of Quercetin on BC (Fig. 7). We experimentally identified that Quercetin could inhibit CYP3A4 to suppress the metabolization of AA to EETs, thereby preventing nuclear translocation of p-Stat3 from alleviating BC in vivo and in vitro.

Our findings initially revealed that Quercetin could inhibit BC cells’ growth and weaken their ability to adhere, migrate, and invade the adjoining cells. Quercetin has various biological effects, including anti-inflammatory, anti-diabetic, antimicrobial, and anti-cancer activities (Reyes-Farias and Carrasco-Pozo 2019). Particularly, accumulating evidence has demonstrated the tumor-suppressive potential of Quercetin in BC. For instance, Khorsandi et al. reported the ability of Quercetin to induce the necroptosis and apoptosis of MCF-7 BC cells (Khorsandi et al. 2017). Our study also revealed that Quercetin at 1 μM concentration exerted the most significant inhibitory effect on cell viability. Another study has reported that Quercetin exerts little function on cell proliferation at concentrations less than 0.7 μM. Co-treatment of doxorubicin and Quercetin suppresses cell proliferation and invasion (Li et al. 2013). Sai et al. elicited the effect of Quercetin in combination with curcumin to inhibit triple-negative BC via regulating various tumor inhibitor genes (Kundur et al. 2019). Additionally, Jia et al. proposed that Quercetin had a restrictive effect on the development of BC by restraining glycolysis and cell mobility through autophagy induction mediated by the Akt-mTOR pathway (Jia et al. 2018). The above findings supported Quercetin's suppressive properties regarding BC cells’ adhesive, migrative, and invasive capacities.

Subsequently, our findings elicited the inhibitory effect of Quercetin on CYP3A4 activity. In consistency with our finding, Himanshu et al. stated that Quercetin could potently and competitively inhibit CYP3A4 in human liver microsomes with a Ki value of 4.12 μM (Rastogi and Jana 2014). Interestingly, inhibition of CYP3A4 suppresses cellular proliferation and enhances the sensitivity to 4-hydroxytamoxifen in TAMR-MCF-7 cells (Thuy Phuong et al. 2017). Then, by a series of experiments, we validated that CYP3A4 regulated AA, 14,15-EET, p-Stat3, and nuclear translocation to aggravate the intensity BC. Existing literature suggests the functionality of AA as a type of bioactive fatty acid released from the membrane lipids as several phospholipase A2 forms (Keith and Miller 2013). AA could radically metabolize to eicosanoids by mediating the cyclooxygenase, lipoxygenase, and CYP pathways to stimulate BC progression (Basu et al. 2013).

Moreover, the AA cascade represents the enzymatic transformation of AA via the CYP and several pathways into biologically active metabolites (Korotkova and Lundberg 2014). Interestingly, as a type of AA metabolite, EETs exhibit vasodilatory, angiogenic, anti-inflammatory, and anti-apoptotic properties (Karkhanis et al. 2017). Furthermore, EET production is closely linked with BC development (Thuy Phuong et al. 2017). Notably, 14, 15-EET is a vital lipid signaling molecule for mediating tumor metastasis and the epithelial-mesenchymal transition of BC cells (Luo et al. 2018). Accumulating evidence has demonstrated the functionality of Stat3 as a potential cytoplasmic transcription factor responsive to protein tyrosine kinase oncoproteins and cytokine signaling via nuclear translocation upon tyrosine-phosphorylation (Gough et al. 2009). Peculiarly, Stat3 often serves as a therapeutic biomarker or target for various tumors (Segatto et al. 2018). The phosphorylation of Stat3 at Tyr 705 could principally activate Stat3 and induce its transcriptional activity via nuclear translocation (Segatto et al. 2018). Besides, CYP-regulated biosynthesis of AA epoxides, explicitly EETs, accelerates tumor growth by facilitating angiogenesis and stimulates the proliferation of tumor epithelia (Guo et al. 2018). Further, CYP3A4 could support the growth of BC cells by inducing nuclear translocation of p-Stat3 (Tyr-705) due to elevated levels of 14, 15-EETs (Mitra et al. 2011). We also investigated the reversal role of exogenously added 14,15-EET in Quercetin and validated the inverse relationship between Quercetin and 14,15-EET. Altogether, our in vitro experiment demonstrated the suppressive role of Quercetin in CYP3A4 to alleviate BC through down-regulating EETs and consequently to prevent the nuclear translocation of p-Stat3.

## Conclusion

To further validate the roles of the proposed mechanism in vivo, we established BC xenograft mouse models. As a result, we found that an appropriate amount of Quercetin could effectively and safely reduce the tumor size and volume. In conclusion, this study proposes the anti-BC activity of Quercetin and supports its use in treating BC (Fig. 7). As a natural product, Quercetin induces fewer side effects and is safer for treating BC patients. However, the dosage should be further optimized, and a better administration would increase the practical value of its clinical application. Moreover, the application of natural medicine in tumor treatment is limited and requires more research, which is our next work.

### Supplementary Information

**Additional file 1: Figure S1. **Effects of saffron extract on apoptosis and viability of breast cancer cells. (A) Flow cytometry was used to detect the apoptosis of ZR-751 breast cancer cells treated with saffron extract at different concentrations; (B) MTS assay was used to determine the viability of breast cancer cells treated with different concentrations of saffron extract. * represents P < 0.05 compared to 0 μg/mL group, *** represents P < 0.001, **** represents P < 0.0001.

**Additional file 2: Figure S2. **Crocetin, isorhamnetin, kaempferol, and Quercetin affect the cytotoxicity of BC MCF-7 and MDA-MB-231 cells at different concentrations. The effect of Crocetin (A), isorhamnetin (B), kaempferol (C), Quercetin (D), and n–n-heptanal (E) on the cytotoxicity and viability of BC MCF-7 and MDA-MB-231 cells was detected by MTS assay. (F) The cytotoxic effect of Quercetin on human normal breast cells MCF10A and MDA-kb2 cells was determined with an MTS assay.

**Additional file 3: Figure S3. **Analysis of the main signaling pathway of five major components of saffron in breast cancer. (A) Network relationship diagram of the target point; (B) Network relationship diagram of the target point based on BC, CC, De screening; (C) Network relationship diagram of the signaling pathway and target genes; (D) Key target gene interaction diagram of Quercetin's anti-cancer effect on breast cancer.

**Additional file 4: Figure S4. **Analysis of the main signaling pathways of the 5 major components of saffron in breast cancer. (A-F) Network relationship diagram of potential target genes for cluster analysis.

## Data Availability

The datasets generated and analyzed during the current study are available in the manuscript and supplementary materials.

## Abbreviations

BC: Breast carcinoma
CTD: Comparative toxicogenomics database
CYP3A4: Cytochrome P450 family 3 subfamily A member 4
EET: Epoxyeicosatrienoic acid
ER: Estrogen receptor
HE: Hematoxylin–eosin
